# Neurodegeneration Biomarkers in Epilepsy: A Critical Review of Current Findings

**DOI:** 10.3390/life16020296

**Published:** 2026-02-09

**Authors:** Ambra Butera, Simona D’Antoni, Antonio Gennaro Nicotera, Maria Vincenza Catania, Carla Consoli, Graziana Ceraolo, Giulia Spoto, Maria Ludovica Albertini, Gabriella Di Rosa, Maurizio Elia

**Affiliations:** 1 Unit of Child Neurology and Psychiatry, Department of Chemical, Biological, Farmaceutical & Environmental Science, University of Messina, 98125 Messina, Italy; butera.ambra@gmail.com; 2 Institute for Biomedical Research and Innovation, National Research Council of Italy (CNR IRIB), via Paolo Gaifami n°18, 95126 Catania, Italy; simona.dantoni@cnr.it (S.D.); mariavincenza.catania@cnr.it (M.V.C.); 3 Unit of Child Neurology and Psychiatry, Maternal-Infantile Department, University of Messina, 98125 Messina, Italy; antonionicotera@ymail.com; 4 Unit of Child Neurology and Psychiatry, Department of Human Pathology of the Adult and Developmental Age “Gaetano Barresi”, University of Messina, 98125 Messina, Italy; carlaconsoli@hotmail.com (C.C.); marialudovica.albertini@gmail.com (M.L.A.); 5 Unit of Child Neurology and Psychiatry, Department of Biomedical Sciences, Dental Sciences & Morphofunctional Imaging, University of Messina, 98125 Messina, Italy; giulia.spoto27@gmail.com (G.S.); gabriella.dirosa@unime.it (G.D.R.); 6 Department of Medicine and Surgery, Kore University of Enna, 94100 Enna, Italy; melia@oasi.en.it; 7 Oasi Research Institute—IRCCS, Via Conte Ruggero 73, 94018 Troina, Italy

**Keywords:** developmental and epileptic encephalopathies, epilepsy, neurodegeneration, neurofilaments light chain, glial fibrillar acid protein, α-synuclein, ubiquitin C-terminal hydrolase L1, tau protein, amyloid-β

## Abstract

Developmental and epileptic encephalopathies (DEEs) represent the most severe group of epilepsies, characterized by drug-resistant seizures, frequent occurrence of epileptiform activity, developmental delay or cognitive impairment. Recent insights have reframed DEEs from static, genetically determined conditions to dynamic disorders with complex and evolving pathophysiology. Several recent studies suggest a link between epilepsy and neurodegeneration, with increased neuronal excitability preceding seizures in conditions characterized by abnormal protein aggregation. Neurodegeneration, defined as the selective and progressive loss of neuronal cells leading to cognitive and functional decline, underlies many progressive neuronal diseases. Although neurodegeneration biomarkers are widely recognized as markers of disease progression in adults, their role in children is still poorly defined and at times controversial. This narrative review aims to summarize current knowledge on the relationship between epilepsy and neurodegeneration, with a focus on potential biomarkers and their implications for disease mechanisms and progression.

## 1. Introduction

Developmental and epileptic encephalopathies (DEEs) represent the most severe group of epilepsies, characterized by drug-resistant seizures, frequent occurrence of epileptiform activity, developmental delay and/or cognitive impairment [1,2]. Typically emerging in early life, DEEs are associated with high rates of mortality and morbidity [1,3]. Advances in genetic sequencing techniques have identified over 400 implicated genes, encompassing numerous age-specific epileptic syndromes, with characteristic EEG patterns and evolving clinical features [1,4,5,6,7].

While acquired epilepsies (i.e., secondary to hypoxic–ischemic injury, trauma, or metabolic insult) are clearly associated with neurodegeneration [4], increasing evidence suggests that DEEs, traditionally considered static neurodevelopmental disturbances, are also dynamic conditions characterized by evolving pathophysiological mechanisms that parallel neurodegeneration [2,8,9]. These findings challenge the traditional dichotomy between non-progressive (neurodevelopmental) and progressive (neurodegenerative) encephalopathies, supporting a continuum where disrupted development and progressive neuronal loss coexist and interact [6,9].

Phenotypic variability within and across DEE-associated genes underscores a shared pathogenic architecture and pronounced pleiotropy, with some conditions potentially arising from neurodegenerative changes processes that begin in utero [9].

Neurodegeneration refers to the progressive loss of neuronal cells leading to cognitive and functional decline [10,11]. While neurodegeneration biomarkers are well established in the elderly populations, their role remains less defined in other age groups, particularly in pediatric populations [6,12,13,14,15,16,17].

Moreover, beyond structural and functional mechanisms, metabolic factors may also influence cognitive outcomes in epilepsy [18]. This review aims to summarize current knowledge on the relationship between epilepsy and neurodegeneration, with a focus on potential biomarkers and their implications for disease mechanisms and progression. We present the biomarkers that currently attract the greatest interest in the study of epilepsy and neurodegeneration, providing an overview of the latest findings in the field.

### Epilepsy and Neurodegeneration

Several recent studies suggested a link between epilepsy and neurodegeneration, with increased neuronal excitability preceding seizures in conditions characterized by abnormal protein aggregation [19]. Biomarkers commonly associated with neurodegeneration, have also been detected in epilepsy, suggesting shared mechanisms and a potential role in monitoring disease progression and therapeutic response, particularly in drug-resistant forms [20,21,22,23]. These observations underscore the conceptual shift in viewing DEEs as entities potentially driven by neurodegenerative processes, including synaptic dysfunction, mitochondrial impairment, and abnormal protein accumulation. Key neurodegenerative pathways, such as those implicated in tauopathies, synucleinopathies, and autophagy defects, have been identified in DEE patients [24,25].

Several genetic mutations linked to DEE further support this connection. Mutations in genes involved in synaptic function (i.e., *CDKL5*, *STXBP1*, *DNM1*) or ion channel regulation (i.e., *SCN1A*, *GRIN2A*) disrupt neuronal communication and contribute to progressive cortical atrophy and cognitive decline. Similarly, genes implicated in neuronal growth and maintenance (*TSC1/TSC2*, *FOXG1*) further highlight the overlap between neurodevelopment and neurodegeneration [24]. Interestingly, certain genetically determined DEEs, particularly those affecting lysosomal and vesicular pathways and intracellular clearance (i.e., *SPTAN1*, *ATP6V1A*, *DMXL2*), suggest neuropathological features resembling adult-onset neurodegenerative disorders, including cortical atrophy, neuronal loss, and protein accumulation [9]. Epileptic seizures may contribute to neuronal and glial damage, triggering a nonspecific neuroinflammatory response [26], although the interplay between inflammation and neurodegeneration in epilepsy remains unclear [10]. One of the key factors linking epilepsy, neuroinflammation and neurodegeneration is microglial activation, which promotes the release of proinflammatory cytokines, such as Tumor necrosis factor α (TNF-α), Interleukin-1β (IL-1β), and Interleukin-6, (IL-6) as well as nitric oxide and reactive oxygen species. These mediators may exert neurotoxic effects, contributing to seizure susceptibility and resistance to drugs [6,26].

Biomarkers of neurodegeneration are measurable indicators of neuronal damage and disease progression. Proteins typically associated with neurodegenerative disease, such as α-synuclein, tau protein, and β-amyloid, have been recently explored in epilepsy [19,20]. Additionally, neuroinflammatory and neuronal injury markers, including cytokines, oxidative stress mediators, glial fibrillary acidic protein (GFAP), ubiquitin C-terminal hydrolase l1 (UCH-L1), and Neurofilaments Light Chain (NfL), have been implicated in seizure-induced neuronal damage [6,17,26,27].

## 2. Materials and Methods

A narrative review of the literature was conducted using PubMed to identify studies exploring the relationship between epilepsy and neurodegeneration. The search strategy included the following keywords and combinations thereof: “developmental AND epileptic encephalopathies AND neurodegeneration,” “epilepsy AND neurodegeneration,” “epilepsy AND neurofilament light chain,” “epilepsy AND glial fibrillary acidic protein,” “epilepsy AND α-synuclein,” “epilepsy AND ubiquitin C-terminal hydrolase L1,” “epilepsy AND tau protein,” and “epilepsy AND amyloid-β.”

Studies were initially screened based on title and abstract, followed by full-text evaluation to assess relevance to the topic of interest. Publications were included if they provided original data or relevant reviews addressing the association between epilepsy and markers of neurodegeneration in pediatric and adult populations, as well as in relevant experimental models. Studies were excluded if they did not specifically address epilepsy-related mechanisms, focused exclusively on non-epileptic neurological disorders, or lacked biomarker-related outcomes. Only studies on idiopathic or genetic epilepsy were included; studies on secondary epilepsies were considered only when included as a comparison group. No restrictions were applied regarding patient age or study design. Additional studies were identified by manually reviewing the reference lists of selected articles.

A total of 138 articles were included in the present review.

## 3. Results

### 3.1. Neurofilaments Light Chain (NfL)

Neurofilaments (Nf) are cytoskeletal proteins that provide structural support to axons. Following neuroaxonal damage, they are released into the cerebrospinal fluid (CSF) and bloodstream [28]. Nf are subdivided into light (NfL), medium (NfM) and heavy (NfH) chain Nf, according to their molecular masses, and are mainly expressed in myelinated axons, where they maintain axonal integrity [29]. Among these, NfL is recognized as promising minimally invasive biomarker of neuronal damage in neurodegenerative disorders and has been extensively studied in both clinical and preclinical contexts [28,29,30,31].

#### NfL and Epilepsy

Elevated NfL levels have been reported in patients with epilepsy of different aetiologies, although its diagnostic and prognostic role remains poorly defined. Increased serum concentrations have been observed immediately after tonic–clonic seizures and in patients with drug-resistant epilepsy [31,32,33,34,35].

Nass et al. measured GFAP and NfL in the serum of 40 adult patients (20 with seizures and 20 healthy controls) and found subtle but statistically significant postictal alterations that normalized within following hours, suggesting that even self-limiting tonic–clonic seizures can induce brain damage [36].

So far, the literature has offered only limited insights into the role of NfL as a biomarker in adults and children with epilepsy [37]. For example, Giovannini et al. conducted a retrospective study including 30 patients with status epilepticus (SE), 30 with drug-resistant epilepsy, and 30 healthy controls, showing that serum NfL levels were significantly elevated in SE, particularly in episodes lasting more than 24 h and in refractory or super-refractory forms. Furthermore, the strong correlation between serum and CSF NfL levels suggests that NfL measurement in the serum may serve as a reliable biomarker of neurological damage, likely due to blood–brain barrier (BBB) disruption during SE [38]. Similar results were obtained in animal models of epilepsy (see below).

Dobson et al. further demonstrated that plasma NfL discriminates epilepsy from psychogenic non-epileptic seizures (PNES) with a positive predictive value of 95%, emphasizing its diagnostic utility [39]. Akel et al. confirmed higher NfL in patients with recent seizures (<2 months) and structural lesions on neuroimaging, especially under 65 years of age. Conversely, no significant difference was found in older patients, underscoring the importance of age adjustment when interpreting NfL values [32].

Additionally, another study by Nass et al. assessed NfL levels in autoimmune epilepsy (AIE), hippocampal sclerosis (HS), genetic generalized epilepsy, and PNES. While NfL was elevated in AIE patients, this was likely influenced by their significantly higher age, as NfL levels correlated strongly with age across all groups. This underlines the necessity of adjusting for age when interpreting NfL values in clinical contexts [40]. In line with this finding, Eriksson et al. reported higher NfL levels in patients with epilepsy or post-stroke epilepsy compared to those who experienced a single seizure [41].

Similarly, Lybeck et al. observed persistent NfL serum elevation up to 72 h after electrographic SE following cardiac arrest, in contrast to GFAP, which did not serve as an independent predictor of clinical outcomes. These findings suggest that NfL reflects neuronal injury, while GFAP reflects glial injury [42].

Additional studies conducted on adult patients confirmed increased NfL levels in drug-resistant epilepsy and prolonged SE [34,43]. Increased serum level of NfL were also found by Giovannini et al. in adult patients with SE compared to those with epilepsy or healthy controls, suggesting that this biomarker could help predict short-term functional outcomes [35].

Ueda et al. found no significant differences in serum NfL levels between patients with epilepsy and controls, although NfL concentrations correlated with cognitive function within the epilepsy group [44]. Similarly, Dargvainiene et al. reported no significant differences in serum levels between epilepsy patients and healthy controls, further highlighting variability in the findings [45]. This apparent discrepancy likely reflects major differences in clinical context and seizure burden between the two cohorts. In the study by Dargvainiene et al., patients mainly experienced isolated or infrequent seizures and were clinically stable at the time of sampling, a condition in which NfL levels may rapidly normalize. In contrast, Giovannini et al. investigated patients with SE, a condition associated with prolonged neuronal injury, BBB disruption, and sustained neuroaxonal damage, which plausibly accounts for the marked NfL elevations observed.

NfL has also been investigated as a prognostic marker in patients with post-anoxic encephalopathy. Disanto et al. found a correlation between these NfL serum levels and the duration of the anoxic insult, reinforcing its predictive value for mortality and its potential as a reliable prognostic marker [46].

In pediatric populations, data are limited. Geis et al. found no significant NfL differences between children with acute neurological events (i.e., meningitis and febrile seizures), chronic neurodevelopmental and neurological conditions (i.e., autism and epilepsy), although a biphasic age pattern was noted, with higher levels in early childhood and adolescence [37]. Similarly, Shahim et al. reported increased NfL, t-tau, and GFAP in children with progressive encephalopathy, epilepsy, or inflammatory CNS disorders compared with controls, with the highest levels in those with SE. They also examined albumin levels hypothesizing that t-tau, NfL, GFAP and the albumin ratio may be altered differently depending on neurological diseases. NfL was higher in patients with infectious and inflammatory CNS disorders, progressive encephalopathy, and tumors compared to those with static encephalopathy. No significant differences were found in the albumin ratio between the groups. Additionally, t-tau and NfL concentrations were elevated in patients with SE compared to those with focal epilepsy and generalized epilepsy. Specifically, patients aged 10–16 years showed higher NfL levels in progressive encephalopathy, infectious and inflammatory CNS disorders, and tumors compared to controls [47].

Consistent with clinical findings, altered NfL expression has been reported in animal models of epilepsy, both in brain tissue [48] and biological fluids (serum/plasma/CSF) [49,50]. In the intra-hippocampal kainic acid mouse model of temporal lobe epilepsy (TLE), NfL levels increased in cerebral interstitial fluid (ISF) and plasma during SE, paralleling the elevations observed in patients [38]. In the chronic phase with spontaneous seizures, NfL remained elevated in ISF and CSF, but not in the plasma [49]. Administration of diazepam and ketamine used to stop SE were related to reduced NfL levels, indicating that plasma NfL reflects acute CNS injury [38].

Similarly, dogs with structural epilepsy due to brain tumors exhibited higher serum NfL compared to those with idiopathic epilepsy, consistent with neuronal injury caused by the tumor [50].

In rats with traumatic brain injury (TBI), plasma NfL increased relative to controls; however, no differences were detected between epileptic and non-epileptic rats, and NfL levels did not correlate with seizure severity. These findings suggest that NfL is a reliable marker of neuronal injury but does not predict post-traumatic epilepsy [51]. Finally, in a kainic acid-induced mouse model of epilepsy, Xin et al. reported dynamic, time-dependent changes in NfL, NfM and NfH expression in brain tissue: levels decreased at 3 h post-seizure induction, rose at 6 h, and declined again at 24 h. These findings indicate a transient disorganization of the neurofilament network related to seizure activity and neuronal injury [52].

Acute elevations are mainly detected within hours to days after generalized tonic–clonic seizures and, most markedly, after SE, whereas interictal levels may rapidly normalize in patients with isolated or infrequent seizures, accounting for the conflicting findings observed in clinically stable versus severe epilepsy cohorts [32]. In contrast, persistently elevated NfL levels independent of recent seizure activity are more suggestive of ongoing neurodegenerative processes rather than seizure-related injury [53]

Overall, these data suggest that NfL levels are elevated in various forms of epilepsy, particularly after tonic–clonic seizures [31,33,36], in drug-resistant epilepsy [32,34], and most markedly in SE supporting its role as a sensitive indicator of neuroaxonal injury associated with seizure activity [38,43].

### 3.2. GFAP

GFAP is a cytoskeletal protein expressed by astrocytic and ependymal cells of the CNS. Reactive astrogliosis, characterized by hypertrophy and proliferation of astrocytes and upregulation of GFAP, occurs in response to neuronal injury, coordinating structural support, cell mobility and signal transduction [53,54,55,56,57,58]. Consequently, elevated GFAP levels in blood and CSF reflect its astrocytic activation upon injury, while physiological blood levels remain low, highlighting specificity for CNS pathology [59,60,61].

#### GFAP and Epilepsy

Although data on GFAP in epilepsy remain limited, available studies suggest that, in adults, GFAP levels are higher in patients with radiologically confirmed epileptogenic lesions and increase with age but show no correlation with sex or disease duration [32,62].

GFAP plasma levels have also been studied as a differentiating marker between epilepsy and PNES, with plasma thresholds above 150 pg/mL demonstrating high specificity [39,63]. In line with these findings, Song and colleagues investigated astrocyte activation in patients with drug-resistant TLE and in a pilocarpine-induced rat model of epilepsy, where they observed an increased expression of GFAP and the P2X7 receptor, accompanied by marked reactive astrogliosis. These results support the hypothesis of persistent astrocyte activation in the chronic phase of epilepsy. However, when interpreting data from pilocarpine-induced models, it should be considered that GFAP upregulation may partly reflect a direct pharmacological effect of pilocarpine on astrocytes rather than seizure-related mechanisms alone, warranting cautious extrapolation to human epilepsy [64].

Mochol et al. conducted a case–control study in which they analyzed serum GFAP levels in 119 adult epilepsy patients and 80 healthy controls, and reported an increase in GFAP in epilepsy patients even after adjusting for confounders (i.e., gender, age, body mass index). These findings suggest astrocyte activation in epilepsy, independently of seizure occurrence, supporting the hypothesis of chronic astrocyte activation in the stable phase of disease [65]. Furthermore, an association between GFAP and autoimmune etiology has been identified. Schulz et al. detected increased GFAP autoantibodies levels in CSF and serum from patients with autoimmune epilepsy versus controls [66].

In pediatric populations, GFAP elevations have been reported in children following generalized tonic–clonic seizures, focal motor seizure, and epileptic spasms with serum levels remaining elevated for months post-seizure and reflecting seizure severity over preceding 6 months [16]. Similar findings have been reported by Aksoy et al. in a cohort of 69 children with refractory and non-refractory epilepsy [67]. Retrospective analyses also show increased CSF GFAP in children with progressive encephalopathy, epilepsy, and CNS inflammatory/infectious disorders versus controls [47].

Gurnett et al., retrospectively analyzed GFAP levels in CSF samples of 52 pediatric patients (aged 5–212 months old) within 24 h of seizure onset. Elevated GFAP levels were observed, particularly in cases of prolonged seizures and symptomatic epilepsy, reinforcing the association between prolonged seizures and astrocyte damage [68].

Wang et al. measured GFAP serum levels in 57 subjects ranging in age from 1 month to 133 months at 1, 4 and 10 days post-SE. GFAP peaks around day 4 post-SE and remains elevated at day 10, suggesting that GFAP may serve as a biomarker for brain injury following convulsive SE [69]. The role of GFAP as a biomarker in epilepsy remains controversial. For example, Mahama et al. evaluated serum GFAP concentrations in 18 adult patients with SE, comparing diazepam-sensitive and diazepam-resistant groups, and found no significant difference between them. These results suggest that GFAP may not reliably predict treatment response in SE, although it could still indicate general astrocytic activation or injury [70]. Similarly, Mayer et al., in an exploratory study of 331 patients with various neurological disorders (including SE), reported no correlation between GFAP plasma levels and SE, implying that while GFAP shows promise in other neurological conditions, it lacks specificity for epilepsy [71].

Preclinical studies corroborate these findings. Increased GFAP expression has been observed in different animal models of epilepsy, including pilocarpine- and kainic acid- induced TLE, mouse models of Dravet and SCN8A encephalopathies, and amygdala-kindled drug-resistant epileptic rats [64,72,73,74,75,76,77,78].

In a mouse model of Dravet, a severe encephalopathy with epilepsy, an increased expression of GFAP and Iba1 (a marker of microglia activation) was detected in both dentate gyrus and entorhinal cortex before occurrence of spontaneous seizures and it was associated with changes in GABAA extrasynaptic receptor composition and GABA tonic currents [75]. An increase in GFAP expression was also observed in a mouse model of SCN8A encephalopathy only after the onset of spontaneous seizures [76,77]. These reactive astrocytes also display a reduction in Kir4.1 channel currents and in glutamine synthetase expression suggesting an impairment in K^+^ ion uptake and in glutamate homeostasis. No changes in microglia expression were detected before or after the onset of seizures in this mouse model [77]. Interestingly, increased expression of GFAP linked to the activation of STAT3 signal transduction pathway was observed in the hippocampus of pilocarpine treated rats after SE, like what occurring in the temporal cortex of patients with drug-resistant epilepsy. Inhibition of STAT3 reduces GFAP upregulation [79], suggesting that STAT3 signal transduction pathway is involved in the increase of GFAP after epilepsy. In the dorsal hippocampus and in the cortex of a rat lithium–pilocarpine model of TLE, the increase in GFAP and Iba1 expression levels at mRNA and protein levels is reduced after a treatment with peroxisome proliferator-activated receptor (PPAR) agonist. This is a pharmacological agent able to inhibit pro-inflammatory processes and activate the protective properties of astrocytes and microglia [80]. These results highlighted that treatments targeting inflammation or specific molecular pathways can modulate the increase of GFAP expression/activation. GFAP may be a marker, but also a potential therapeutic target for epilepsy.

However, not all studies confirm these associations. Some adult and animal studies report no significant correlation between GFAP and epilepsy or seizure frequency [71,81,82]. Overall, available evidence indicates that GFAP alterations in epilepsy primarily reflect astrocytic activation and glial injury, particularly in association with structural lesions and prolonged or severe seizures, supporting its role as a marker of seizure-related astroglial response. All these data are summarized into Table A2.

### 3.3. α-Synuclein

α-synuclein is a small protein (140 amino acids, 14 kDa) predominantly expressed in the CNS encoded by the SNCA gene. Under physiological conditions, α-synuclein plays a crucial role in synaptic function being involved in neurotransmitter vesicle trafficking and synaptic plasticity and modulating synaptic vesicle recycling and neurotransmitter release [83,84]. However, its pathological aggregation into intracytoplasmic inclusions is a hallmark of several neurodegenerative disorders, including Parkinson’s disease, dementia with Lewy bodies, multisystem atrophy, and alcoholism, where it serves as a biochemical prognostic marker [85].

#### α-Synuclein and Epilepsy

The major pathophysiological mechanism linking α-synuclein to epileptogenesis involves mitochondrial dysfunction. The accumulation of misfolded α-synuclein leads to Lewy bodies formation in susceptible neurons, primarily within the basal ganglia, and disrupts mitochondrial integrity affecting voltage-dependent anion channels. This results in ionic imbalance across neuronal membranes, further aggravated by oxidative stress, elevated intracellular Ca^2+^ levels, and increased proinflammatory cytokines, all contributing to aberrant neuronal hyperexcitability. These alterations trigger a vicious cycle of oxidative stress, membrane lipid peroxidation, glial activation, and neuroinflammation, ultimately leading to neurodegeneration and increased excitability [19,86,87,88].

All these findings highlight a molecular link between epilepsy and major neurodegenerative disorders, suggesting shared pathogenic mechanisms and potential therapeutic targets [19]. However, further investigations are required to clarify whether inhibiting α-synuclein aggregation could provide therapeutic benefits in epilepsy [6].

While α-synuclein has been extensively studied in age-related neurodegenerative disorders, research in epilepsy remains limited [89]. Increasing evidence links α-synuclein aggregation to neural hyperexcitability and seizure susceptibility. Both direct and indirect observations suggest its involvement in epilepsy: patients with duplications and triplications of *SNCA* frequently experience seizures [90]. The role of α-synuclein has also been investigated in drug-resistant epilepsy, characterized by recurrent seizures and intracellular deposits of α-synuclein. Rong et al. analyzed total α-synuclein levels in serum and CSF in 67 epileptic patients (aged between 17 and 68 years), finding significantly higher concentrations in drug-resistant cases, whereas no significant differences were found in newly diagnosed or drug-responsive epilepsy [85]. Choi et al. conducted a prospective study measuring serum and exosomal α-synuclein in 115 children with epilepsy and 10 with acquired CNS demyelinating disorders, compared to 146 controls. Both patient groups showed significantly elevated serum α-synuclein levels compared to controls correlating with disease severity in both conditions [89]. Similarly, Zheng and Kong reported higher α-synuclein levels in 110 children with epilepsy versus controls, with a positive correlation between α-synuclein concentration, EEG abnormality severity, and electroencephalographic discharge indices [91].

In another case–control study, Salem et al. measured plasma α-synuclein in 60 children with idiopathic epilepsy and 30 healthy controls, finding significantly higher levels in patients, particularly in those with generalized seizures. α-synuclein levels were also correlated with seizure frequency and duration, reinforcing its potential role as a marker of epilepsy severity [92].

At the tissue level, Zhang et al. analyzed α-synuclein expression in brain samples from 15 patients with focal cortical dysplasia type IIb (FCD IIb), 24 with tuberous sclerosis complex (TSC), and 20 controls. They observed reduced α-synuclein protein levels in both FCD IIb and TSC samples compared to controls, despite increased *SNCA* mRNA in TSC, suggesting post-transcriptional dysregulation. Consistent findings were reported in FCD rat models generated by in utero X-ray radiation, showing decreased α-synuclein mRNA and protein levels, but increased phosphorylated α-synuclein immunoreactivity in cortical lesions. These changes were associated with enhanced α-synuclein-NMDA receptor interaction, suggesting a role in synaptic transmission modulation [93].

Additional studies in human and animal models further support these findings. Yang et al. described an aberrant expression of cytoskeletal and synaptosomal proteins, including reduced α-synuclein expression in hippocampal tissue from adult patients with mesial TLE, suggesting synaptic and cytoskeletal impairments [94]. Conversely, Li et al. found increased α-synuclein expression in a pilocarpine-induced mouse model of TLE [95]. The analysis of oligomeric and phosphorylated α-synuclein forms is therefore recommended in both patients and experimental models to better clarify its functional role [85].

Overall, available evidence indicates that altered α-synuclein levels in epilepsy are primarily associated with seizure severity and drug-resistant forms, supporting a role in synaptic dysfunction and protein homeostasis imbalance [93,95].

All these findings are summarized into Table A3.

### 3.4. Ubiquitin C-Terminal Hydrolase L1

Ubiquitin C-terminal hydrolase L1 (UCH-L1), also known as PGP9.5, is a neuron-specific cytoplasmic enzyme and one of the most abundantly expressed proteins in neurons, accounting for 1–5% of total soluble neuronal proteins [16,35,96]. Lower expression occurs in gonadal tissues, fibroblasts during wound healing and certain clonal cell lines. Despite its abundance, it is not involved in neuronal development, but is essential for maintaining axonal integrity [16,97].

UCH-L1 plays a crucial role regulating the ubiquitin–proteasome system (UPS), a critical mechanism for maintaining protein homeostasis within the ATP-dependent proteasomal pathway. It functions primarily as a deubiquitinating enzyme that hydrolyzes ubiquitin C-terminal esters and amides [98,99], but also exhibits ubiquitin ligase activity, with α-synuclein among its substrates [96,97]. Functional studies demonstrate that UCH-L1 contributes to synaptic function [100] and exerts neuroprotective effects against hypoxic–ischemic injury [101]. Indeed, its role in neurodegenerative disease pathophysiology has gained increasing attention [98,99].

Due to its neuronal specificity and minimal peripheral expression, UCH-L1 serves as a valuable biomarker for neuronal injury. Elevated CSF and blood levels indicate neuronal damage and have been linked to neuroinflammation, neurodegeneration, traumatic brain injury, and BBB disruption [16,35,97,102].

In Alzheimer’s disease (AD), increased CSF UCH-L1 and its co-localization with ubiquitin in amyloid plaques and neurofibrillary tangles implicate UPS dysfunction in disease pathogenesis [99]. Conversely, in Parkinson’s disease, reduced blood UCH-L1 levels have been observed in patients with severe cognitive decline, indicating that decreased protein levels may reflect neuronal damage associated with cognitive impairment [98].

#### UCH-L1 and Epilepsy

While the role of UCH-L1 in neurodegenerative diseases has been well explored [98,99], its involvement in epilepsy remains unclear [67].

In human studies, Mondello et al., measured plasma and CSF UCH-L1 levels in 52 adults with epilepsy (17 with a cryptogenic focal or idiopathic epilepsy) admitted to the emergency department after single or recurrent tonic–clonic or focal to bilateral tonic–clonic seizures, compared with 19 neurologically healthy controls. They found significantly higher UCH-L1 concentrations in both CSF and plasma within the first 12 h post-seizure compared to the controls. However, plasma levels decreased over time, underscoring the importance of early sampling for accurate detection [102].

Similarly, Li et al. measured UCH-L1 in CSF samples of 33 patients with epilepsy (aged 17–60) presenting with seizures and found significantly elevated levels within 24 h post-seizure, particularly in generalized and repetitive seizures. UCH-L1 concentrations correlated positively with both seizures’ duration and severity [103].

Yasak et al. also reported higher serum UCH-L1 levels in adult epilepsy patients compared to healthy individuals, though no significant difference was observed between seizure and interictal periods [96].

In line with this, Asadollahi and Simani found significantly elevated serum UCH-L1 in patients with epilepsy compared to individuals with PNES and healthy controls, though no correlation emerged between UCH-L1 levels and seizure type [104].

In the pediatric population, available data are even more limited. Elhady et al. observed elevated serum UCH-L1 levels in children with epilepsy, particularly those with active seizures and generalized-onset epilepsy [16]. Conversely, Aksoy et al. found no significant differences between children with non-refractory epilepsy, refractory epilepsy, and healthy controls [67].

Animal studies provide additional insights. In a kindling model of epilepsy, plasma UCH-L1 levels increased significantly following seizure activity, suggesting that seizure-induced neuronal damage can be reflected by serum measurement [81]. Interestingly, in a kainic acid-induced SE model, UCH-L1 was found to be down-regulated in the mouse hippocampus (specifically CA3 region) 24 h post-SE, and pharmacological inhibition of UCH-L1 prolonged seizures and exacerbated neuronal death, supporting its neuroprotective role [105]. Similarly, in a pentylenetetrazole-induced rat model, the intraperitoneal co-injection of a UCH-L1 inhibitor increased seizure severity [106].

Overall, available evidence indicates that UCH-L1 alterations in epilepsy are primarily associated with acute seizure activity and correlate with seizure duration and severity [16,96,102,103], supporting its role as a marker of activity-dependent neuronal injury and protein homeostasis imbalance [16,97]. These findings are summarized into Table A4.

### 3.5. Tau Protein and Neurofibrillary Tangles

Tau is a microtubule-associated protein encoded by the MAPT gene, on chromosome 17 [107]. It is primarily localized in neurons of the CNS, particularly in axons, but can also be found in somatodendritic compartments, nuclei, glial cells and, to a lesser extent, the synaptic cleft [107,108]. Tau plays a crucial role in microtubule stabilization, axonal integrity, and intracellular transport, functions tightly regulated by phosphorylation [6,109]. 

Beyond its structural function, tau participates in several intracellular signaling pathways, including PI3K–Akt–GSK3β, MAPK, and mTOR pathways, together with reduced PP2A activity, promotes tau hyperphosphorylation, neurofibrillary tangle (NFT) formation, and neuronal dysfunction [110,111]. Hyperphosphorylation decreases tau’s affinity for tubulin, disrupting microtubule stability, while caspase-mediated cleavage further enhances aggregation into NFTs, emphasizing the contribution of cell death pathways to tauopathy pathogenesis [109]. Under pathological conditions, neuronal injury leads to increased CSF levels of total tau (t-tau) and phosphorylated tau (p-tau) [107,108,112]. 

Elevated CSF tau has been documented in multiple neurodegenerative and acute neurological disorders, including traumatic brain injury, ischemic stroke, viral encephalitis, and Creutzfeldt-Jakob disease, supporting its role as a biomarker of neuroaxonal degeneration [6,107,112]. Given its primarily intracellular localization, tau serves as a valuable biomarker for axonal injury and neuronal degeneration [107,108,112].

#### Tau, NFTs and Epilepsy

Recently, there has been a growing interest in the role of tau protein in epilepsy. Neurological disorders characterized by tau accumulation, such as AD and post-traumatic encephalopathies, exhibit a higher incidence of epilepsy, suggesting a bidirectional relationship between tauopathies and epilepsy [6,107]. 

However, clinical studies examining CSF tau levels in patients with epilepsy have yielded conflicting results. Palmio et al. analyzed CSF samples from 54 patients with tonic–clonic or focal to bilateral tonic–clonic seizures collected within 48 h post-seizure and found no significant differences in t-tau and p-tau levels between idiopathic or cryptogenic epilepsy. However, increased tau concentrations were observed exclusively in patients with acute or previously occurred symptomatic seizures. Tau levels did not correlate with seizure frequency, occurrence of SE, or sampling interval [112]. Similarly, Monti et al. analyzed t-tau and p-tau CSF levels in 28 patients (aged 11 to 79 years) with SE and found higher CSF t-tau levels in those with refractory compared to drug-responsive patients. CSF t-tau correlated positively with SE duration, disability risk, and chronic epilepsy, indicating its potential as a biomarker of SE severity and prognosis [108]. 

Conversely, Shahim et al. reported lower CSF t-tau and p-tau levels in adult patients with epilepsy compared to healthy controls, with no significant differences among seizure subtypes [113]. 

Fonseca et al. investigated multiple CSF biomarkers and amyloid Positron Emission Tomography (PET) findings (using 18F-flutemetamol) in 30 adult patients with drug-resistant TLE. Elevated p181-tau levels were detected in 7% of patients and correlated with poorer performance on verbal fluency tests, suggesting a potential link between p-tau accumulation and cognitive impairment in TLE patients [114].

Tai et al. examined cortical resections from 33 adults with drug-resistant TLE and observed hyperphosphorylated tau (hp-tau) pathology, including neuropil threads and NFTs, in 94% of samples, linking chronic epilepsy with tau related neurodegeneration and cognitive decline [14]. Subsequent studies largely confirmed these findings [115,116], though not all replicated them [117]. More recently, Aroor et al. found NFT and non-tangle p-tau structures in 50% of temporal cortex biopsies from 12 adults with refractory epilepsy, with phosphorylation at Thr205 and Thr181 correlating with epilepsy duration. They also identified a positive association between p-tau and phosphorylated S6 (p-S6) at Ser235/246, implicating mTOR pathway dysregulation in hp-tau [118].

Tau levels have also been measured in blood. Akel et al. analyzed plasma tau in 204 epileptic adults and found no significant correlation between tau levels, age, or epilepsy type, suggesting limited diagnostic value in peripheral samples [32].

Consistent with human data, animal models demonstrate seizure-induced hp-tau. In a kainic acid-induced TLE model, Canet et al. found persistent p-tau accumulation in both epileptogenic and contralateral hippocampus, indicating that seizure activity alone can trigger AD-like tau pathology [119]. Similarly, Liu et al. showed reduced phosphatase 2A activity an p-tau accumulation in epileptogenic regions (amygdala, hippocampus, and cortex) across different epilepsy models [13], while Alves et al. observed increased tau and p-tau within 24 h post-SE, predominantly in microglia, emphasizing cell-specific and temporal differences between acute and chronic epilepsy [120].

Interestingly, mechanistic studies suggest a causal contribution of tau to epileptogenesis and seizure-related cognitive dysfunction. In Kcna1 knockout mice, reducing tau expression decreased hyperexcitability, seizure frequency, and mortality [121]. Similarly, Gao et al. showed that optogenic stimulation of CA1 neurons induced seizures and production of hp-tau, while targeted tau proteolysis reduced seizures and improved cognition, highlighting tau as a potential therapeutic target [122].

Overall, available evidence indicates that tau alterations in epilepsy are associated with seizure chronicity, drug-resistance, and cognitive impairment, supporting a role for phosphorylation-related network dysfunction and cumulative neuronal stress in epileptic brain pathology. The biological significance of hyperphosphorylated tau detected in cortical tissue from patients with epilepsy remains unclear. In the absence of clinical dementia, such findings should not be interpreted as evidence of a primary tauopathy, and their long-term functional consequences remain to be elucidated [13,108,118,121,122]. All these data are summarized into Table A5.

### 3.6. Amyloid-β and Amyloid Precursor Protein

Amyloid-β (Aβ) is a peptide derived from the cleavage of the amyloid precursor protein (APP) by β- and γ-secretase. Aβ accumulation represents a key pathological hallmark of AD, promoting neurodegeneration through the formation of toxic extracellular plaques, both diffuse and neuritic, and by facilitating intraneuronal NFTs formation [123].

APP is a transmembrane protein composed of a large extracellular and a smaller intracellular domain. Beyond its role in Aβ generation, full-length APP exerts essential physiological functions, contributing to synaptic regulation, neuronal survival, and the modulation of GABAergic neurotransmission [124].

APP processing occurs via two major pathways: the non-amyloidogenic and amyloidogenic routes. In the non-amyloidogenic pathway, cleavage by α-secretase generates soluble APP-alpha (sAPPα), thus precluding Aβ formation. Conversely, the amyloidogenic pathway involves initial cleavage by β-secretase (BACE1) to produce soluble APP-beta (sAPPβ), followed by γ-secretase-mediated cleavage, leading to the production of Aβ peptides [115]. Variability at the γ-secretase cleavage site results in peptides with different C-terminal lengths, including Aβx-38, Aβx-40, and Aβx-42 [113]. Because of its central role in the generation of neurotoxic Aβ aggregates, APP remains a major focus of investigation in AD and other neurodegenerative conditions [6].

#### Aβ, APP and Epilepsy

While Aβ is a well-established hallmark of AD pathology, emerging evidence suggests its involvement in epileptogenesis, although its pathogenic significance in epilepsy remains debated. Human studies support an association between Aβ and epilepsy. Fonseca et al. reported significant Aβ accumulation in mesial temporal and anterior cingulate regions of patients with TLE, with 23% showing abnormally low CSF Aβ1–42 levels [114]. Conversely, Tai et al. identified Aβ plaques in only 5 of 33 TLE patients, with no correlation with cognitive performance, whereas tau pathology was linked to cognitive impairment (see previous paragraph) [14]. These findings suggest that plaque pathology is uncommon and poorly correlated with clinical outcomes in most epilepsy cohorts.

Increased APP expression has also been implicated in epilepsy-related neurodegeneration. Sheng et al. found β-APP-positive neuronal accumulation in hippocampal and cortical layers III-V, suggesting a potential marker for seizure-induced neuronal damage [125]. Similarly, Sima et al. observed elevated β-APP protein levels despite unchanged APP in mRNA, supporting its role in neuronal network injury associated with epilepsy [12].

Gourmand et al. further demonstrated elevated APP, BACE1 activity, and neurotoxic soluble Aβ species, with Aβ56 being significantly upregulated both in the hippocampus and temporal cortex, whereas Aβ42 was selectively increased in the hippocampus of drug-resistant TLE patients, indicating seizure-driven alterations in APP processing rather than primary amyloidogenic dysregulation. APP upregulation correlated with synaptogenesis and neurite outgrowth, potentially promoting network hyperexcitability. They also identified activation of c-Jun N-terminal kinase, associated with APP phosphorylation and BACE1 upregulation. In the same cohort, higher Aβ levels correlated negatively with cognitive performance, suggesting a possible contribution of amyloid dysregulation in cognitive deficits [115].

However, other studies yielded inconsistent findings [117,118]. Silva et al. detected moderate Aβ plaque deposition in only 4 of 56 TLE patients with minimal correlation to psychometric test scores, indicating that epilepsy-related cognitive decline may occur independently of amyloid plaque pathology [117]. Similarly, Aroor et al. found Aβ deposits in 8 of 12 drug-resistant epilepsy samples, but no association with cognition, age or epilepsy duration was detected. The weak correlation with mTOR activation suggests independent pathological mechanisms [118]. Joutsa et al., using Pittsburgh compound B (PiB) PET, observed abnormal amyloid uptake in 22% of adults with childhood-onset epilepsy, particularly in apolipoprotein E (APOE) ε4 carriers, indicating a potential interaction between genetic susceptibility and epilepsy-related mechanisms of Aβ deposition [126].

In contrast, Shahim et al. found no overall CSF Aβ differences, but increased Aβx-38, Aβx-40, and Aβx-42 in patients with repetitive focal seizures, linking Aβ release to synaptic activity and excitotoxicity [113].

Experimental models and clinical evidence further support that soluble Aβ species enhances seizure susceptibility and excitotoxicity, often preceding plaque formation [127,128]. Transgenic AD mouse models expressing mutant APP and presenilin-1 display spontaneous seizures [127,129,130], while Aβ1–42 oligomer application induces neuronal hyperexcitability and seizure predisposition [131], though not consistently replicated [132].

All these findings suggest that Aβ and APP alterations in epilepsy are primarily associated with dysregulated APP processing, accumulation of soluble Aβ species, and activity-dependent network remodeling, supporting a role in excitotoxic stress and maladaptive synaptic plasticity within epileptic brain circuits. All these data are summarized into Table A6 and Table A7.

## 4. Discussion

Current research increasingly focuses on identifying reliable biomarkers of epilepsy diagnosis, monitoring the progression of the disease, and assessing treatment response. Emerging evidence supports a link between epilepsy and neurodegeneration, challenging the traditional view of drug-resistant epilepsy as a static condition [2,9]. Several biomarkers classically associated with neurodegenerative disease, NfL, GFAP, α-synuclein, UCH-L1, tau, and Aβ/APP, show alterations in different epileptic contexts and may reflect seizure-induced neuronal injury, chronic network dysfunction, and neuroinflammatory activation [28,85]. 

Among these, NfL is the biomarker with the most consistent findings: it increases after tonic–clonic seizures and is markedly elevated in SE [32,38]. These data support its value as an indicator of acute neuronal injury, although interpretation remains strongly age-dependent [39]. GFAP reflects astrocytic activation and is frequently elevated in patients with structural brain lesions or prolonged seizures [32,65]. However, results remain heterogeneous and its specificity for epilepsy is limited [70]. α-synuclein shows increased serum or CSF levels in both pediatric and adult patients with epilepsy, particularly in drug-resistant epilepsy [85,89], suggesting a role in neuronal stress and abnormal protein homeostasis, although tissue studies indicate etiology-specific dysregulation [93]. From a genetic perspective, *SNCA* duplications or triplications are well-established causes of synucleinopathies; however, seizures have been reported only in a subset of affected individuals and current evidence does not support a direct or quantifiable association with epilepsy [90]. In tissue studies, Tau protein alterations, including hyperphosphorylation and NFT formation, reflect those observed in tauopathies and are linked to cognitive decline in TLE, reinforcing its neurodegenerative nature [6,14,89]. 

Conversely, CSF and plasma tau levels show variable changes across studies and the mechanisms driving tau aggregation remain unclear [32,112]. pTau and Aβ accumulation in drug-resistant epilepsy even without clinical dementia, further supports shared molecular pathways with neurodegeneration, possibly involving mTOR dysregulation [118]. Altered APP processing and accumulation of Aβ species have been identified in hippocampal and temporal regions of TLE patients [115], while plaque deposition appears rare and inconsistently related to cognitive profiles highlighting a complex interaction between amyloid dysregulation, seizure burden, and cognitive decline [14,114,117]. UCH-L1 has been recognized as a sensitive marker of acute brain injury, yet its precise role in epilepsy is still debated [35,97,102].

Before discussing the implications of individual biomarkers, it may be helpful to clarify the conceptual framework used to interpret biomarker changes in epilepsy. Particularly, a distinction can be made between biomarkers reflecting acute seizure-related neuronal or glial injury and those potentially indicative of chronic or progressive neurodegenerative processes. However, this distinction is often context-dependent rather than absolute in epilepsy. In fact, several biomarkers discussed in this review (i.e., NfL, GFAP, tau, UCH-L1) may show transient increases following acute seizures or SE [32,36,38,42,68], whereas persistent elevations or tissue-level alterations may be associated with cumulative neuronal injury, network remodeling, or neurodegenerative mechanisms [6,14,89,115,118].

**Figure A1:**
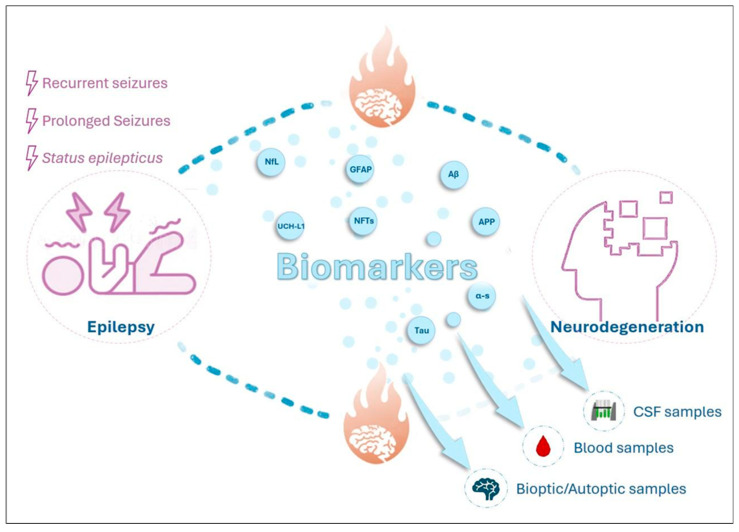
**Schematic representation of the bidirectional relationship between epilepsy and neurodegeneration.** In the upper left section, epileptic conditions responsible for the release of pro-inflammatory cytokines and neurodegeneration biomarkers are illustrated. At the center, the main biomarkers studied in the context of epilepsy and neurodegeneration are shown. In the lower right section, the tissues and biological fluids (serum, cerebrospinal fluid, and anatomical or autoptic samples) in which these biomarkers can be measured are indicated.

Despite neuroinflammation being an integral component of the epilepsy-neurodegeneration continuum, it is not the primary focus of the present review; instead, these biomarkers highlight the convergence of neuroinflammatory, excitotoxic, and protein-aggregation pathways in epilepsy and underscore their potential as accessible biomarkers for disease characterization and longitudinal assessment [6,36,70] (Figure A1).

Experimental models have replicated several of these findings. SE induced by pilocarpine or kainic acid reproduces seizure-driven tau phosphorylation, α-synuclein aggregation, and neuroinflammation, while transgenic models (*Kcna1^−/−^*, Dravet, *SCN8A*) demonstrate synaptic dysfunction and impaired autophagy [76,121]. However, these approaches—typically relying on acute-insult protocols in young rodents—fail to mirror the chronic, age-dependent progression of human DEEs, as they tend to capture seizure-related neuronal damage rather than the long-term processes of epileptogenesis, network remodeling, and progressive dysfunction. Future studies should combine humanized *in vivo* models with patient-derived in vitro systems, such as pluripotent stem cells (iPSC)-derived neurons and cortical organoids and benefit from standardized sampling protocols and shared biobanks to strengthen translational reliability. Despite growing evidence supporting a link between epilepsy and neurodegeneration, significant gaps remain in understanding the underlying molecular mechanisms partly due to substantial heterogeneity in biological samples, analytical methods, and population stratification across studies [133,134].

Variability exists in analytical platforms, biological matrices, and sampling time points relative to seizure occurrence, which complicates cross-study comparisons. A major interpretative limitation concerns the timing of biomarker sampling: most human studies rely on cross-sectional designs and single post-ictal measurements, which inherently preclude a reliable discrimination between seizure-induced damage and primary neurodegenerative processes. This limitation is methodological rather than biomarker-specific and applies consistently across neuronal and glial markers. Consequently, current evidence does not allow a reliable discrimination between seizure-induced damage and primary neurodegenerative processes. Longitudinal interictal sampling, standardized temporal windows, and multimodal integration with imaging and electrophysiology will be essential to overcome this constraint. Publication bias may further contribute to overestimation of biomarker consistency and effect size. In addition, differences in epileptic phenotypes (i.e., isolated seizures versus SE), along with variability in drug-resistance and antiseizure treatments, are often underreported or uncontrolled. This may substantially influence biomarkers findings, particularly for markers sensitive to astrocytic and inflammatory activation such as GFAP [39,63,92].

To date, no biomarker reliably quantifies disease progression or neurodegenerative risk. Among those discussed, NfL appears the most promising candidate for longitudinal assessment, given its relative stability, correlation between CSF and blood levels, and association with cumulative neuronal injury [32,38,39]. Nonetheless, its age dependence and sensitivity to acute seizure events require cautious interpretation, while the limited specificity of GFAP constrains its use as a standalone longitudinal marker. Indeed, changes in circulating biomarker levels may reflect not only neuronal injury but also astrocytic dysfunction and alterations in BBB integrity. Discrepancies between markers (i.e., elevated NfL in the absence of GFAP changes, as reported by Lybeck et al.) may thus reflect disease-specific and analytical factors rather than true biological dissociation [32,65,70]. Tau, α-synuclein, UCH-L1, and amyloid-related biomarkers show greater variability and appear more closely linked to acute neuronal stress or tissue-level pathology, limiting their current applicability for routine long-term disease monitoring [85,89,97,102,112]. Multimodal panels combining neuronal and glial markers may therefore provide more robust insights.

In this context, alongside fluid biomarkers, advanced neuroimaging techniques may provide complementary insights into neurodevelopmental and neurodegenerative processes in epilepsy. Quantitative susceptibility mapping (QSM), an MRI-based technique sensitive to tissue magnetic properties related to iron and myelin content, has demonstrated potential capturing age-dependent microstructural and degenerative changes, although their clinical translation remains limited [135].

Clinical monitoring therefore continues to rely on seizure frequency, or treatment response, EEG findings, imaging, and, when possible, standardized neuropsychological assessments [28,136]. Accordingly, fluid biomarkers should be interpreted as complementary tools that gain clinical relevance only when integrated into a multimodal assessment. Further exploration of inflammatory mediators, such as IL-1β, TNF-α, and IL-10 remains critical, given their key role in excitotoxicity and neuronal damage.

In the pediatric cohort, available evidence remains limited and often heterogenous. Studies investigating serum and CSF biomarkers such as α-synuclein, UCH-L1, GFAP, and NfL in children with epilepsy have reported inconsistent findings, frequently constrained by small sample sizes and methodological variability [27,32,35,67]. Age-dependent factors may profoundly influence biomarker kinetics and interpretation [37,70]. Furthermore, the absence of standardized reference ranges and age-adjusted cutoffs hampers comparability between pediatric and adult populations. Normal brain development is characterized by an early phase of intense synaptogenesis followed by activity-dependent synaptic pruning that progressively refines cortical connectivity across childhood and adolescence [137]. During these developmental stages, physiological synaptic remodeling and glial maturation may contribute to biomarker variability, even in the absence of overt neurodegenerative processes. Experimental evidence suggests that altered pruning mechanisms may also play a role in developmental epileptogenesis, underscoring the need to consider developmental stage-specific biology when interpreting pediatric biomarkers [138]. Future longitudinal studies, stratified by developmental stage, are therefore essential.

In conclusion, the clinical value of epilepsy biomarkers will depend on their integration with neuroimaging, electrophysiology, and neuropsychological assessment [2,91]. Considering the BBB alterations induced by prolonged seizures [38], blood-based biomarkers offer a practical and translationally viable approach. Identifying robust and reproducible peripheral markers could transform epilepsy management, from symptomatic control to personalized, disease-modifying neuroprotective strategies.

## Data Availability

No new data were created or analyzed in this study. Data sharing is not applicable to this article.

## Abbreviations

The following abbreviations are used in this manuscript:

Aβ	Amyloid-β
AD	Alzheimer’s disease
AI	Autoimmune
AIE	Autoimmune Epilepsy
APOE	Apolipoprotein E
APP	Amyloid Precursor Protein
BACE1	β-Secretase (Beta-Site APP-Cleaving Enzyme 1)
BBB	Blood–Brain Barrier
CNS	Central Nervous System
CSE	Convulsive Status Epilepticus
CSF	Cerebrospinal Fluid
DEEs	Developmental and Epileptic Encephalopathies
DRE	Drug Resistant Epilepsies
EEG	Electroencephalography
FBTCS	Focal to Bilateral Tonic–Clonic Seizure
FCD	Focal Cortical Dysplasia
FCD IIb	FCD Type IIb
GABA	Gamma-Aminobutyric Acid
GGE	Genetic General Epilepsy
GFAP	Glial Fibrillary Acidic Protein
GTCS	Generalized Tonic–Clonic Seizure
Hp-tau	Hyperphosphorylated Tau
HS	Hippocampal Sclerosis
IL-1	Interleukin-1β
IL-6	Interleukin-6
IL-10	Interleukin-10
iPSC	Induced Pluripotent Stem Cells
ISF	Cerebral Interstitial Fluid
MRI	Magnetic Resonance Imaging
nSE	Non-Convulsive Status Epilepticus
Nf	Neurofilaments
NfH	Neurofilament Heavy Chain
NfL	Neurofilament Light Chain
NfM	Neurofilament Medium Chain
NFT	Neurofibrillary Tangle
PET	Positron Emission Tomography
PiB	Pittsburgh Compound B
PNES	Psychogenic Non-Epileptic Seizures
p-tau	Phosphorylated Tau
sAPPβ	Soluble Amyloid Precursor Protein Beta
SE	Status Epilepticus
sNfL	Serum Neurofilament Light Chain
TBI	Traumatic Brain Injury
TLE	Temporal Lobe Epilepsy
TNF-α	Tumor Necrosis Factor α
t-tau	Total tau
TSC	Tuberous Sclerosis Complex
UCH-L1	Ubiquitin C-Terminal Hydrolase L1
UPS	Ubiquitin–Proteasome System

## Appendix A

**Table A1:** Data of the studies evaluating NfL levels in Epileptic subjects.

Species	Population	Type of Sample	Results	Epileptic Features	Detection Method	Additional Information	Reference
*Homo* *sapiens*	28 Epi adults (21 CSE vs. 7 nSE) vs. 1186 HC	CFS, serum	↑ NfL in SE (*p* = 0.001) n.s. differences between CSE and nSE	SE	Simoa	NfL concentrations in CFS and serum showed a high correlation (*p* < 0.001)	Margraf et al. [43]
*Homo* *sapiens*	13 AIE vs. 13 HS vs. 7 GGE vs. 8 PNES	CSF, serum	n.s. in GGE, ↑ NfL in AIE in serum and CSF	F or G seizures	Simoa	-	Nass et al. [40]
*Homo* *sapiens*	60 Epi adults (30 SE vs. 30 DRE) vs. 30 HC	Serum (+CFS in subset)	↑ serum NfL in SE pts vs. DRE (*p* < 0.001) and HC (*p* < 0.001). n.s. differences between DRE and HC	SE and DRE	Simoa	CFS NfL levels strongly correlate with serum (τ = 0.68, *p* < 0.001)	Giovannini et al. [38]
*Homo* *sapiens*	46 Epi adults (last seizure < 6 months) vs. 49 Epi adults (seizure free >1 year)	Serum	n.s. differences	F or G seizures or unknown seizures	Simoa	NfL levels were increased in male patients, mainly within age-adjusted reference ranges.	Dargvainiene et al. [45]
*Homo* *sapiens*	38 Epi adults vs. 24 HC	Serum	n.s. differences (*p* = 0.92)	F or G seizures or unknown seizures	ELISA	Correlation between NfL and cognitive levels	Ueda et al. [44]
*Homo* *sapiens*	87 SE vs. 30 Epi adults vs. 27 HC	Serum	↑ NfL in SE (*p* < 0.001)	SE	Immunoassay	Samples collected during SE (≤72 h); serum NfL predicted 30-day outcome	Giovannini et al. [35]
*Homo* *sapiens*	20 Epi adults vs. 20 HC	Serum	↑ NfL in epi adults pts	F or G seizures	Simoa	-	Nass et al. [36]
*Homo* *sapiens*	104 Epi adults vs. 22 PNES vs. 12 NED	Plasma	↑ NfL in Epi pts compared to PNES (*p* = 0.04)	NA	Simoa	-	Dobson et al. [39]
*Homo* *sapiens*	204 Epi adults	Plasma	↑ NfL in pts with seizures ≤ 2 mo (*p* = 0.006). ↑ NfL in younger pts with epileptogenic lesions (*p* < 0.001)	NA	Simoa	Higher NfL levels in focal vs. generalized epilepsy (<65 years)	Akel et al. [32]
*Homo* *sapiens*	117 Epi pts vs. 79 HC children	CSF	↑ NfL in epilepsy (*p* < 0.001) and progressive encephalopathy ↑ NfL in SE compared to focal epilepsy (*p* < 0.01), primary generalized epilepsy (*p* < 0.001) and unspecified epilepsy group (*p* < 0.01)	F or G seizures or unknown seizures, SE	Immunoassay	-	Shahim et al. [47]
*Homo* *sapiens*	6 Epi children vs. 34 febrile controls vs. 23 ANC vs. 37 FS vs. 18 CNC vs. 3 SSD vs. 101 HC	Serum	n.s. differences in sNfL levels	n.a.	Simoa	-	Geis et al. [37]

**Legend:** ↑: increased; ANC: Acute Neurologic Conditions; CNC: Chronic Neurologic Conditions; Epi: epileptic, F: Focal; FS: Febrile Seizures; G: Generalized; HC: Healthy Controls; n.a.: not available; NED: non-epileptic disorders; n.s.: not significant; pts: Patients; Simoa: Single Molecule Array; SSD: Severe Systemic Disease.

**Table A2:** Data of the studies evaluating GFAP levels in Epileptic subjects.

Species	Population	Type of Sample	Results	Epileptic Features	Detection Method	Additional Information	Reference
*Homo* *sapiens*	13 AIE vs. 13 HS vs. 7 GGE vs. 8 PNES adults	CSF, serum	n.s. differences	F or G seizures	Simoa	-	Nass et al. [40]
*Homo* *sapiens*	119 Epi adults vs. 80 HC	Serum	↑ GFAP in Epi pts (*p* = 0.042)	F or G seizures	ELISA	-	Mochol et al. [65]
*Homo* *sapiens*	20 Epi adults vs. 20 HC	Serum	↑ GFAP in Epi adults pts (*p* < 0.001 a postictal peak at 125.3 pg/mL)	F or G seizures	Simoa	-	Nass et al. [36]
*Homo* *sapiens*	43 SE vs. 20 PNES vs. 19 HC adults	Serum	↑ GFAP in pts with SE (*p* < 0.001) n.s. differences between PNES and HC (*p* = 0.5)	F or G seizures/SE	ELISA	-	Simani et al. [63]
*Homo* *sapiens*	104 Epi adults vs. 22 PNES vs. 12 NED	Plasma	↑ GFAP in Epi pts compared to PNES (*p* < 0.04)	Various epilepsy subtypes (including DRE)	Simoa	-	Dobson et al. [39]
*Homo* *sapiens*	204 Epi adults	Plasma	↑ GFAP in pts with seizures ≤2 mo (*p* = 0.032). ↑ GFAP in younger pts with epileptogenic lesions (*p* < 0.001)	F or G seizures or unknown seizures, SE	Simoa	-	Akel et al. [32]
*Homo* *sapiens*	331 adults ND including SE (n. n.a.)	Plasma	n.s. differences	SE	Immunoassay	↑ GFAP in SE (n.s.)	Mayer et al. [71]
*Homo* *sapiens*	12 SE DRV vs. 6 SE DRT adults	Peripheral blood	n.s. differences	n.a.	ELISA	Blood collected ≤72 h post-seizure	Mahama et al. [70]
*Homo* *sapiens*	25 Epi adults vs. 6 HC	Temporal cortex resection specimens	↑ GFAP immunoreactivity in resected tissue from TLE patients (*p* < 0.01)	TLE	IHC and Western blot	-	Song et al. [64]
*Homo* *sapiens*	52 Epi children vs. 33 HC	CSF	↑ GFAP in Epi pts (*p* = 0.0075) (correlation with seizure duration)	F or G seizures	ELISA	CSF collected ≤24 h post-seizure	Gurnett et al. [68]
*Homo* *sapiens*	117 Epi children vs. 79 HC	CSF	↑ GFAP in Epi pts (*p* < 0.1)	F or G seizures or unknown seizures, SE	Immunoassay	Biomarkers distinguish progressive from static disorders	Shahim et al. [47]
*Homo* *sapiens*	35 RE vs. 18 NRE vs. 16 HC children	Serum	↑ GFAP in Epi pts (*p* = 0.001)	F or G seizures	ELISA	-	Aksoy et al. [67]
*Homo* *sapiens*	57 CSE vs. 30 HC children (1,4,10 th day)	Serum	↑ GFAP in pediatric pts with CSE (4th day) (*p* < 0.01)	G epilepsy and CFS	ELISA	-	Wang et al. [69]
*Homo* *sapiens*	30 Epi children vs. 30 HC	Serum	↑ GFAP in Epi pts (*p* < 0.0001)	F or G seizures	ELISA	GFAP correlated with seizure severity in the past 6 months; high levels predicted active seizures (*p* = 0.035)	Elhady et al. [16]
*Mus* *musculus*	75 kindle mice vs. 32 control rats	Plasma	n.s. differences	n.a.	ELISA	-	Chmielewska et al. [81]
*Rattus norvegicus*	84 pilocarpine-treated vs. 6 control rats	Hippocampal tissue	↑ GFAP expression in the hippocampus of rats with spontaneous recurrent seizures	TLE	IHC, WB and RT-qPCR	HC: Non-epileptic controls (post-traumatic tissue)	Song et al. [64]

**Legend:** ↑: increased; DRT: Diazepam-Resistant; Epi: epileptic; F: focal; G: generalized; HC: Healthy controls; IHC: immunohistochemistry; n.a.: not available; ND: Neurological diseases; NED: non-epileptic disorders; NRE: Non refractory epilepsy; n.s.: not significant; pts: patients; RE: Refractory Epilepsy; RT-qPCR: Reverse Transcription quantitative Polymerase Chain Reaction; Simoa: Single Molecule Array; WB: Western blot.

**Table A3:** Data of the studies evaluating α-synuclein levels in Epileptic subjects.

Species	Population	Type of Sample	Results	Epileptic Features	Detection Method	Additional Information	Reference
*Homo* *sapiens*	40 DRE vs. 14 TE vs. 13 Epi adults newly diagnosed vs. 22 HC	CSF, serum	↑ α-synuclein in DRE (*p* < 0.05)	F or G seizures	ELISA	HC: pts with neurosis	Rong et al. [85]
*Homo* *sapiens*	110 Epi children vs. 35 HC	Serum	↑ α-synuclein in Epi pts (*p* < 0.05)	F or G epilepsy	ELISA	Positive correlation with EEG abnormalities	Zheng & Kong [91]
*Homo* *sapiens*	30 DRE vs. 30 WCE vs. 30 HC children	Serum	↑ α-synuclein in Epi pts (*p* < 0.001) ↑ α-synuclein in DRE vs. WCE (*p* < 0.001)	F or G epilepsy	ELISA	α-Syn negatively correlated with time since last seizure and age at onset (*p* = 0.001, 0.016)	Salem et al. [92]
*Homo* *sapiens*	115 Epi children vs. 10 acquired demyelinating disorders vs. 146 HC	Serum (and serum-derived exosomes)	↑ α-synuclein in Epi pts (serum levels related with disease severity) (*p* < 0.05) ↑ α-synuclein in pts with demyelinating disorders (*p* < 0.05)	F or G seizures	ELISA	Blood ≤48 h post-seizure; exosomal α-syn correlated with serum (*p* < 0.0001).	Choi et al. [89]
*Homo* *sapiens*	15 FCD IIb and 24 TSC children with DRE vs. 26 HC	CTX	↓ α-synuclein mRNA in FCD IIb, (*p* < 0.01) ↑ α-synuclein mRNA in TSC (*p* < 0.05) ↓ α-synuclein protein in FCD IIb (*p* < 0.05) ↓ α-synuclein protein in TSC (*p* < 0.01)	F epilepsy associated with FCD IIb/TSC	IHC, IF, Western blotting, Co-immunoprecipitation, RT-PCR	-	Zhang et al. [93]
*Mus musculus*	58 prenatal X-ray–induced FCD rat model	CTX	↓ α-synuclein mRNA in FCD (*p* < 0.01) ↓ α-syn immunoreactivity in FCD (*p* < 0.05) ↑ p-α-syn protein in FCD (*p* < 0.01–0.001) ↓ p-α-syn immunoreactivity in FCD (*p* < 0.01) ↓ α-synuclein protein in FCD (*p* < 0.05)	-	IHC, IF, Western blotting, Co-immunoprecipitation, RT-PCR	Analyses were performed in rats at postnatal days 7, 14, and 28	Zhang et al. [93]

**Legend:** ↑: increased; ↓: decreased; CTX: cortex; Epi: epileptic; F: focal; G: generalized; HC: Healthy controls; IF: Immunofluorescence; IHC: Immunohistochemistry; pts: patients; RT-PCR: Reverse Transcription Polymerase Chain Reaction; TE: tractable epilepsy; WCE: Well-controlled epilepsy.

**Table A4:** Data of the studies evaluating UCH-L1 levels in Epileptic subjects.

Species	Population	Type of Sample	Results	Epileptic Features	Detection Method	Additional Information	Reference
*Homo* *sapiens*	33 Epi adults vs. 23 HC	CSF	↑ UCH L1 post-seizure	F or G seizures	ELISA	UCH-L1 levels were higher in G than in F seizures, and showed no correlation with age or epilepsy-related clinical variables	Li et al. [103]
*Homo* *sapiens*	52 Epi adults	CSF, plasma	↑ UCH-L1 post-seizure	GTCS (Single or repetitive) or FBTCS	ELISA	Plasma UCH-L1 levels decreased with increasing time to sampling; CSF and plasma levels were strongly correlated and associated with age in epileptic patients, but not in controls	Mondello et al. [102]
*Homo* *sapiens*	160 Epi adults vs. 100 HC	Plasma	↑ UCH-L1 in Epi adults	ES and nSP	ELISA	No difference in UCH-L1 between epileptic seizure and non-seizure periods	Yasak et al. [96]
*Homo* *sapiens*	43 Epi adults vs. 20 PNES vs. 19 HC	Serum	↑ UCH-L1 post-seizure	F or G seizures	ELISA	-	Asadollahi and Simani [104]
*Homo* *sapiens*	35 RE vs. 18 NRE vs. 16 HC children	Serum	n.s. in UCH-L1	SeLFE, F seizures, IGE, DEEs, CP, NMD or TSC	ELISA	-	Aksoy et al. [67]
*Homo* *sapiens*	30 Epi children vs. 30 HC children	Serum	↑ UCH-L1 in Epi children	F or G seizures	ELISA	UCH-L1 levels were higher in G seizures than in F seizures and were significantly elevated in children with active seizures compared to those seizures-free for the previous 6 months	Elhady et al. [16]
*Mus musculus*	43 kindled mice vs. 32 control mice	Plasma	↑ UCH-L1 post-seizure	PTZ induced	ELISA	-	Chmielewska et al. [81]
*Mus musculus*	Preconditioned (tolerance) vs. sham-preconditioned (injury)	Hippocampal tissue samples	↓ UCH-L1 in hippocampus 24 h after KA induced SE	F-onset SE KA induced	WB	-	Reynolds et al. [105]
*Mus musculus*	Kindled PTZ mice vs. control mice	Hippocampal tissue samples	↑ UCH-L1 Epi mice	PTZ and LDN-57444 induced	WB, IF, IP, and Timm staining	LDN-57444 treatment increased seizure severity in PTZ-kindled mice, with higher Racine score, shorter latency, and more stage 4–5 seizures	Wen et al. [106]

**Legend:** ↑: increased; ↓: decreased; CP: cerebral palsy; ELISA: enzyme-linked immunosorbent assay; Epi: epileptic; ES: epileptic seizures; F: focal; G: generalized; HC: healthy controls; IF: Immunofluorescence; IGE: Idiopathic generalized epilepsy; IP: Immunoprecipitation; KA: kainic acid; NMD: Neuronal migration disorder; NRE: Non refractory epilepsy; n.s.: not significant; nSP: non-seizure period; PTZ: Pentylenetetrazole; RE: Refractory epilepsy; SeLFE: Self-limited focal epilepsy; WB: Western blot.

**Table A5:** Data of the studies evaluating tau levels in Epileptic subjects.

Species	Population	Type of Sample	Results	Epileptic Features	Detection Method	Additional Information	Reference
*Homo* *sapiens*	30 Epi adults	CSF	n.s. in t-tau and p-tau in different seizures type	TLE	CLEIA	↑ t-tau and ↑ p-tau in patients with poorer performance in verbal fluency	Fonseca et al. [114]
*Homo* *sapiens*	28 adolescents and adults with SE	CSF	↑ t-tau in 14 pts ↑ p-tau in 6 pts	SE	ELISA	t-tau levels were higher in pts who developed a RSE and positively correlated with SE duration; elevated CSF t-tau was associated with increased risk of developing disability and chronic epilepsy	Monti et al. [108]
*Homo* *sapiens*	54 Epi adolescents and adults vs. 31 HC	CSF	↑ t-tau and ↑ p-tau post-seizure in pts with acute or remote symptomatic seizures	GTCS or FBTCS	ELISA	No statistical differences in t-tau or p-tau levels between different etiologic group or HC	Palmio et al. [112]
*Homo* *sapiens*	45 Epi adults vs. 17 HC	CSF	↓ t-tau and p-tau vs. HC	F or G (single, repetitive, or nSE)	ELISA	-	Shahim et al. [113]
*Homo* *sapiens*	204 Epi adults	Plasma	n.s. in t-tau in different epilepsy type	F, G or unknown epilepsy	Simoa	n.s. differences in plasma t-tau between patients with recent seizures and those who were seizure-free	Akel et al. [32]
*Homo* *sapiens*	12 Epi adults	TL samples	NTs and NFTs as forms of p-tau structures in half biopsies	DRE	ELISA	Robust presence of p-tau (Ser202/Thr205)-related neuropil threads and neurofibrillary tangles in epilepsy biopsies, with no significant correlation between p-tau Thr205 and Thr181	Aroor et al. [118]
*Homo* *sapiens*	19 Epi adults vs. 9 Alzheimer cases vs. 22 HC	TL and/or hippocampal tissue samples	↑ tau5 in TLE hippocampus (*p* < 0.01) but not in temporal cortex	TLE	WB and IHC	tau5 (*p* < 0.05) and p-tau AT180 (*p* < 0.05) were negatively correlated with executive function; Aβ42 levels correlated with tau 4R in TLE cortex (*p* < 0.05)	Gourmaud et al. [115]
*Homo* *sapiens*	56 Epi adults	TL and hippocampal tissue samples	NFTs and p-tau in TL of 2/56 pts	Drug-resistant TLE	IHC	-	Silva et al. [117]
*Homo* *sapiens*	33 Epi adults	TL samples	NFTs, NTs, and pre-NFTs as forms of p-tau in TLE	TLE	IHC	Tau pathology extent correlated with decline in verbal learning (r = 0.63), recall (r = 0.44), and GNT scores (r = 0.50) over 1 year post-TL resection (*p* ≤ 0.05).	Tai et al. [14]
*Homo* *sapiens*	22 Epi adults vs. 20 post-mortem control	TL and hippocampal tissue samples	p-tau (95%), mature NFTs (66.7%), and pretangles (100%) in sclerotic hippocampi of Epi adults	MTLE	WB and IHC	↑ p-tau leveled were associated with impaired BNT (*p* = 0.033) and Digit Span Forward (*p* = 0.048) scores	Toscano et al. [116]
*Mus musculus*	112 mice	TL samples	↑ p-tau	KA induced	ELISA	-	Canet et al. [119]
*Mus musculus*	18 genetically modified mice (ChR2-expressing excitatory neurons in ventral CA1)	Ventral CA1 hippocampal tissue samples	↑ p-tau and ↑ t-tau in hippocampus and cortex after repeated seizures	Optogenetically induced seizures	WB and IHC	C4 treatment ↓ p-tau and ↓ tau, ameliorated seizure severity (increased latency, reduced duration), and improved spatial memory	Gao et al. [122]
*Mus musculus*	19 *Kcna1^−/−^Tau^−/−^* mice vs. 15 *Kcna1^−/−^Tau^+/+^* mice vs. 15 WT *Tau^+/+^* mice	Hippocampal slices	Tau loss in *Kcna1^−/−^* mice ↓ burst frequency and duration to WT levels; *Kcna1^−/−^, Tau^+/+^* mice showed high burst frequency; no effect in WT slices	*Kcna1^−/−^* mice	In vitro electrophysiology (hippocampal slice recordings)	-	Holth et al. [121]
*Mus musculus*	60 mice	AMY, HIP, and CTX	↓ PP2A ↑ p-tau in key epileptogenic brain regions	AK, KA SE, and PTE	BCA kit	-	Liu et al. [13]

**Legend:** ↑: increased; ↓: decreased; +: plus; <: minor; >: major; AK: Amygdala kindling; AMY: Amygdala; BCA: Bicinchoninic acid protein assay; CLEIA: Chemiluminescent Enzyme Immunoassay; CTX: cortex; ELISA: enzyme-linked immunosorbent assay; F: focal; G: generalized; GNT: graded naming test; HC: healthy controls; IHC: Immunohistochemistry; HIP: hippocampus; KA: kainic acid; MTLE: Mesial temporal lobe epilepsy; n.s.: not significant; NTs: Neuropil Threads; PP2A: phosphatase 2A activity; PTE: Post-Traumatic Epilepsy; pts: patients; RSE: Refractory Status Epilepticus; TL: temporal lobe; WB: Western blot; WT: wild-type.

**Table A6:** Data of the studies evaluating Aβ levels in Epileptic subjects.

Species	Population	Type of Sample	Results	Epileptic Features	Detection Method	Additional Information	Reference
*Homo* *sapiens*	45 Epi adults vs. 17 controls *	CSF	↑ Aβx-38, Aβx-40 in rPS vs. nSE (*p* < 0.01), sPS & controls (*p* < 0.05) ↑ Aβx-42 in rPS vs. nSE (*p* < 0.05) sAPP: n.s.	F or G (single, repetitive or nSE)	ELISA	-	Shahim et al. [113]
*Homo* *sapiens*	30 Epi adults	CSF and PET imaging	↑ Aβ PET uptake in MTL bil (*p* < 0.001), ipsi > ant cingulate (*p* = 0.020) ↓ CSF Aβ1–42 in 7 pts (23%) CSF Aβ1–42 and other brain areas on Aβ PET: n.s.	TLE	PET and CLIA	-	Fonseca et al. [114]
*Homo* *sapiens*	41 Epi adults vs. 46 matched HC	Brain PiB-PET imaging	↑ PiB uptake: higher in Epi cases than controls (*p* = 0.04)	Unspecified COES	PET	↑ PiB uptake in cases strongly associated with the APOE genotype	Joutsa et al. [126]
*Homo* *sapiens*	12 Epi adults	TL samples	Robust Aβ plaques but n.s. correlation to cognitive performance	Drug-resistant epilepsy	IHC and ELISA	-	Aroor et al. [118]
*Homo* *sapiens*	56 Epi adults	TL samples	Aβ plaques have been found just in 4/56 pts n.s.	Drug-resistant TLE	IHC	pts with Aβ plaques showed lower scores on verbal memory assessment scales	Silva et al. [117]
*Homo* *sapiens*	33 Epi adults	TL samples	Aβ plaques have been found just in 5/33 pts n.s.	Drug-resistant TLE	IHC	n.s. correlation was found between Aβ plaque deposition and cognitive performance (*p* = 0.02)	Tai et al. [14]

**Legend:** *: suspicion of headache or central nervous system infectious; ↓: decreased; ↑: increased; ant: anterior; bil: bilaterally; CLIA: Chemiluminescence immunoassay; COES: Childhood-onset Epilepsy Syndrome; Epi: Epileptic; HC: healthy controls; IHC: Immunohistochemistry; ipsi: ipsilateral; MTL: mesial temporal lobes; n.s.: Not significant; PET: positron emission tomography; PiB: amyloid ligand carbon 11–labeled Pittsburgh Compound B; pts: patients; rPS: repetitive partial seizures; sAPP: soluble amyloid precursor protein; TL: temporal lobe.

**Table A7:** Data of the studies evaluating APP levels in Epileptic subjects.

Species	Population	Type of Sample	Results	Epileptic Features	Detection Method	Additional Information	Reference
*Homo* *sapiens*	19 Epi adults vs. 9 Alzheimer cases vs. 22 HC	TL and/or hippocampal samples	↑ β-APP and Aβ42 in the hippocampus (*p* < 0.01) ↑ pAPP in hippocampus and temporal cortex (*p* < 0.01) ↑ BACE1 and soluble Aβ56 in both the hippocampus (*p* < 0.05) and temporal cortex (*p* < 0.0001)	TLE	WB and IHC	pAPP (*p* < 0.05), BACE (*p* = 0.001), tau5 (*p* < 0.05), and P-tau AT180 (*p* < 0.05) were negatively correlated with executive function + correlation between Aβ42 and tau 4R in TLE cortex (*p* < 0.05) + correlation between BACE1 and full-length APP in both TLE hippocampus (*p* < 0.05) and temporal cortex (*p* < 0.05)	Gourmaud et al. [115]
*Homo* *sapiens*	36 Epi adults vs. 25 controls *	TL and/or hippocampal samples	↑ β-APP in TL and hippocampal sample of cases (*p* < 0.05) APP mRNA concentrations: n.s.	Unspecified refractory epilepsy	qPCR, IHC and IF	No differences in APP mRNA concentrations between the epileptic and control groups	Sima et al. [12]
*Homo* *sapiens*	8 Epi adults vs. 8 HC	TL samples	↑ β-APP 770 and 751 (*p* < 0.05) of cases β-APP 695: n.s. ↑ β-APP-positive neurons in TL (*p* < 0.001) of cases ↑ IL-1α in microglia in case (*p* < 0.001)	TLE	WB and IHC	-	Sheng et al. [125]

**Legend:** *: brain trauma or herniation due to cerebral hemorrhage; ↑: increased; +: positive; Aβ42: amyloid-β42; Aβ56: amyloid-β56; β-APP: β-amyloid precursor protein; HC: healthy controls; IF: Immunofluorescence; IHC: Immunoistochemistry; IL-1α: Interleukin-1α; n.s.: Not significant; pAPP: phosphorylated APP; qPCR: quantitative Polymerase Chain Reaction; sAβ56: soluble Aβ56; TL: temporal lobe.
